# Nursing Home Post-Acute Care Specialization Groups and Financial Outcomes

**DOI:** 10.1093/geroni/igab046.935

**Published:** 2021-12-17

**Authors:** Xiao (Joyce) Wang, Jeffrey Burr, Robert Weech-Maldonado, Jennifer Hefele, Kathrin Boerner

**Affiliations:** 1 Brown University, Providence, Rhode Island, United States; 2 McCormack Graduate School, Boston, Massachusetts, United States; 3 University of Alabama at Birmingham, Birmingham, Alabama, United States; 4 Booz Allen Hamilton, McLean, Virginia, United States; 5 University of Massachusetts Boston, Boston, Massachusetts, United States

## Abstract

Nursing homes (NHs) have increasingly specialized in post-acute care (PAC). However, it remains unclear as to why some NHs engage in more specialization than the others. Furthermore, the relationship between financial outcomes and PAC specialization has not been examined using more accurate financial indicators. This study developed a NH PAC specialization typology and examined financial outcomes (i.e. total revenue per inpatient day, operating margin) of different specialization groups. We employed NH-level panel data from 2011 through 2017 and focused on over 9,000 urban NHs per year. Multiple data sources were utilized like the Certification and Survey Provider Enhanced Reporting data; Medicare Cost Reports; and Brown University’s LTCfocUS. We employed Latent Profile Analysis to develop distinct NH care specialization groups based on PAC staffing levels. This analysis revealed heterogeneous and clustered patterns of PAC staffing utilization and identified a four-group typology: “low specialization,” “mixed specialization,” “moderate PAC specialization,” and “intensive PAC specialization.” Using fixed-effects modeling, we then examined financial outcomes of the four PAC specialization groups. Although being in a group with higher level of commitment to PAC specialization was associated with higher revenues, it was not necessarily associated with higher operating margins. Further, in stratified analyses, for-profit and not-for-profit NHs showed different patterns in these associations. This suggested that although NHs compete for patients paid at higher reimbursement policies, increased costs may offset higher revenues as a result of specialization. Future studies should track financial outcome trajectories of NHs by care specialization groups in light of various payment innovations.

